# Supplementary material for: Designing highly efficient interlocking interactions in anisotropic active particles
Source: Nat Commun. 2024 Jul 7;15:5692. doi: 10.1038/s41467-024-49955-x (PMC11227507; doi:10.1038/s41467-024-49955-x)
Supplement: Supplementary file 1 — Supplementary Information [file 41467_2024_49955_MOESM1_ESM.pdf]

Designing highly efficient interlocking interactions in anisotropic active particles - Supplementary Information

Solenn Riedel[1], Ludwig A. Hoffmann[2], Luca Giomi[2], and Daniela J. Kraft[1]

[1]Soft Matter Physics, Huygens-Kamerlingh Onnes Laboratory, Leiden University, PO Box 9504, 2300 RA Leiden, the Netherlands
[2]Instituut-Lorentz, Leiden University, P.O. Box 9506, 2300 RA Leiden, The Netherlands

1 Supplementary Methods

1.1 Overview of experimental samples

Shape		
180° concave-side leading	surface area fraction after 80-90 min	crescents/mm^2
	0.022%	9
	0.039%	15
	0.063%	25
	0.261%	104
90° concave-side leading	surface area fraction after 60-70 min	
	0.013%	7
	0.023%	12
	0.062%	32
	0.080%	42
260° concave-side leading	surface area fraction after 160-220 min	
	0.027%	7
	0.042%	11
	0.077%	21
180° convex-side leading	surface area fraction after 45 or 90 min	
	0.008%	3
	0.018%	7
	0.024%	10
	0.035%	14
straight rods	surface area fraction after 60-80 min	
	0.011%	6
	0.028%	14
	0.059%	29
	0.095%	48

1.2 Dynamic simulations

To simulate the experimental system we follow the model of Wensink et al. [?]. We simulate N self-propelling particles in two dimensions that move with a velocity v_0. We employed the same particle dimensions and velocity in the simulations as in experiments, see below for a mapping between computational and physical units. The dynamics is assumed to be overdamped and particles interact only by steric repulsion with each other, i.e., there is no hydrodynamic interaction. To build a particle it is discretized into $i = 1, ..., k$ equidistant spherical segments, each with diameter d. The diameter is set by the exponential short-range pair potential we use to model the particles as hard spheres. According to the specific opening angle α and radius R of a given particle we distribute the m spheres along a circle segment of radius R (see Fig. 1). Each particle moves with a velocity v_0. A particle ρ has an orientation $\boldsymbol{u}_\rho$ and a position $\boldsymbol{r}_\rho$. The position of segment i with

respect to the position of the center of mass $\boldsymbol{r}_\rho$ is denoted by $\boldsymbol{e}^i_\rho$.

The pair potential of two particles ρ and δ is given by $U_{\rho\delta} = k^{-2}\sum_{i,j=1}^{k} u\left(r^{ij}_{\rho\delta}/d\right)$. Here, $u(x) = u_0 \exp\left(-x\right)/x^2$ is a short-range potential that is repulsive if $u_0 > 0$. This results in effectively hard particles. $r^{ij}_{\rho\delta} = \left|\boldsymbol{r}_\rho - \boldsymbol{r}_\delta + \boldsymbol{e}^i_\rho - \boldsymbol{e}^j_\delta\right|$ is the distance of two segments of the two different particles. The equations of motion, found from balancing forces and torques due to activity and steric repulsion, for a particle ρ are given by [?]:

$$f_t \partial_t \boldsymbol{r}_\rho = F_\mathrm{a} \boldsymbol{u}_\rho - \boldsymbol{\nabla}_{\boldsymbol{r}_\rho} U + \xi \tag{1a}$$

$$f_r \partial_t \varphi_\rho = -\boldsymbol{\nabla}_{\varphi_\rho} U + \eta \tag{1b}$$

where f_t and f_r are translational and rotational friction, respectively, $\boldsymbol{u}_\rho = \{\sin\varphi_\rho, \cos\varphi_\rho\}$, $v_0 = F_\mathrm{a}/f_t$, and $U = \sum_{\rho,\delta(\rho\neq\delta)} U_{\rho\delta}/2$. ξ and η are translational and rotational Brownian noise, respectively.

We simulate the particles in a square system with periodic boundary conditions. A number N particles is initialized in a system of size $D \times D$ with random orientation and random position. We use periodic boundary conditions and choose the system to be the same size as the one used in the experiments (see below). Varying the system size by as much as a factor of 9, we found that the periodicity of the boundary is irrelevant for the clustering dynamics. The dynamic of each particle is given by the equations above.In the simulations we have defined a particles to be in a cluster with another particles if it is closer than $3R$ for 100 iterations. Unless otherwise noted, each of the results presented is found by averaging over 100 independent runs. We use the following parameters for the simulation: $D = 1137$, $v_0 = 0.04$, $d = 0.5$, $u_0 = 1$, $f_t = 1$, $f_r = 1$, $m = 9$, $\xi = 0$, $\eta = 0$. For the 180°-particles we have used $R = 1$. Hence, if a single such particle is initialized in the system of size $D \times D$, the area surface fraction is given by $\phi_s \approx 0.00012\%$. This can be used to easily convert number of particles in the simulations into area surface fraction. For other opening angles we have adapted R according to if arc-length or cross-section is fixed. To map the quantities of the simulations into physical units we used the following experimental values: $v_0 = 1$ μm/s and $R = 4$ μm for a 180° particle. We thus find convert lengths and times from simulation into physical units as follows: 1 length = 4 μm, and 1 time step = 0.16 s.

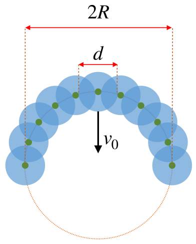

Supplemental Figure 1: **Sketch of particle used in simulation.** A particle of Radius R and opening angle α is constructed by arranging discs of radius d on a spherical segment. The particle moves with a velocity v_0.

We set the translational and rotational noise to zero in the simulations presented in the main text. We have compared simulations including the rotational and translational noises with values measured in the experiments in the experimental setup and compared simulations with and without noise. Including the noise did not change the behavior significantly, see also below.

1.3 Analytical balls-into-bins model

It is possible to model the clustering of the active particles as a simple probabilistic process for which we can also find some analytical expressions and results. In the following, we first run a simulation of a balls-into-bins model that yields results very similar to the experiments and dynamics simulations of the active particles. Second, we can find an analytical expression for the expected number of particles in a cluster for this balls-into-bins model. However, the analytical expression is very complicated and not very insightful. Thus, in the third part, we show that at short times there is a linear regime that corresponds to a Poisson process. The plateauing that is observed at longer times can then be described in this framework as a time-dependent rate that decreases over time.

Simulation of balls-into-bins model We have initially N active particles of cross-sectional length L which move with a speed v_0 in a square system of width and height D. The dynamics of the active particles are mapped onto a balls-into-bins model as follows. The system is divided into boxes of size S; thus there are a total of $B = (D/S)^2$ boxes. The size S of a box is determined by the size of the particle and we set $S \approx L$, assuming for simplicity that that if two active particles are in contact they will form a cluster. In the first step of the simulation we put each of the N particles randomly into one of the boxes. If two (three, ...) balls are assigned to the same box, we count this as a two- (three-, ...)cluster. If a ball is in a box by itself, it is counted as a free particle. In the following step of the simulation the above procedure is repeated but only all the *free* particles are assigned a new box. That is, if in the previous step particles formed a cluster, they are kept in this box and are not randomly assigned a box again. See Fig. 2a for a sketch. This models the purely rotational, and absence of translational, motion of clusters that we observe in the experiments. This procedure is repeated M times in total. If, at some point, a particle is assigned to a box that is already occupied by two (three, ...) particles, this is count this as a three (four, ...) cluster. Note that in this model a cluster, once formed, will never decay. This corresponds to the zero-noise limit and neglects potential collisions between free particles and clusters that can result in the destruction of the clusters, resulting in a finite lifetime. However, these are second-order effect, and, as a first approximation, these can be ignored for the sake of simplicity of this model.

We can convert the steps in the simulations into physical time steps as follows. We approximate that each time step in the simulation occurs after sufficient time has passed for an active particle to move to another box, i.e. each step of the simulation corresponds to a physical time step $\Delta t = S/v_0$. Thus, the total time the simulations run is given by $t_{\text{total}} = MS/v_0$. Some results of the simulations are shown in Fig. 2b. It is easily seen that the shape of the resulting curves is very similar to the ones found in experiment and dynamical simulations, see Fig. 2c. A quantitative agreement can be found by fine-tuning the parameters N, S, and L.

Analytical expression for the simulation To derive a general analytical expression for this balls-into-bins model we consider only two-clusters forming. That is, in the following we ignore the possibility of higher-order cluster forming. This is justified by the observation that two-clusters are dominant (at least at short times and/or low concentrations). In principle, it is straightforward to allow for higher-order clusters in following derivation. However, for the sake of simplicity we opt to consider the simplest case, which we find to agree well with the simulations. That is because the probability of a three-cluster forming in the simulations for $N \ll B$ is small. Indeed, running the above simulation under the same assumption (only two-clusters form) results in a curve very similar to the ones where all cluster sizes are taken into account, see Fig. 2d. Furthermore, we assume that at each time-step of the simulations at most one cluster can form. Again, this is justified by the probability of a cluster forming for $N \ll B$ being very small.

The main difficulty of the analytical derivation is due the probability of a cluster forming is potentially changing at each time step of the simulations because, once a cluster has formed, the number of balls one draws from decreases. Namely, the probability that, for N_f balls (free particles) and B bins, two balls end up in the same bin is given by the "birthday paradox" probability

$$P^{N_f} \equiv P(N_f, B) = 1 - Q(N_f, B) = 1 - \frac{B!}{(B - N_f)! B^{N_f}} \, . \tag{2}$$

As the number of free particle decreases over time, this probability is dependent on the time step as well. Thus, for an initial state of N balls we have one two-cluster after the first step with probability P^N and no cluster with probability Q^N. We can then draw a tree diagram for the following steps, see in Fig. 2e. For every two-cluster forming the number of free particles decreases by two, that is the probabilities become P^{N-2} etc. From this one can compute the expected number of particles in a cluster after T steps. From the tree diagram we find the following expression for the number of particles in a cluster after T steps:

$$N_c(T, N) = \sum_{n=2, n \in 2\mathbb{Z}}^{\min(2T, N)} \left\{ n \left[\prod_{k=0}^{\frac{n}{2}-1} P(N - 2k) \right] \sum_{m_0 + m_1 + \ldots = T - \frac{n}{2}} \prod_{j=0}^{\frac{n}{2}} Q^{2m_j}(N - 2j) \right\} \, . \tag{3}$$

While exact, this expression is computationally very expensive to compute for large T as the number of terms grows exponentially with T. For this reason, we will derive a simplified expression below that is a good approximation for the cases of small density we consider experimentally. Note that Eq. (3) simplifies considerably if the probabilities are assumed to be constant, which is true if $N_c \ll N$. In this case the complicated term (the sum in the last term) just reduces to a binomial

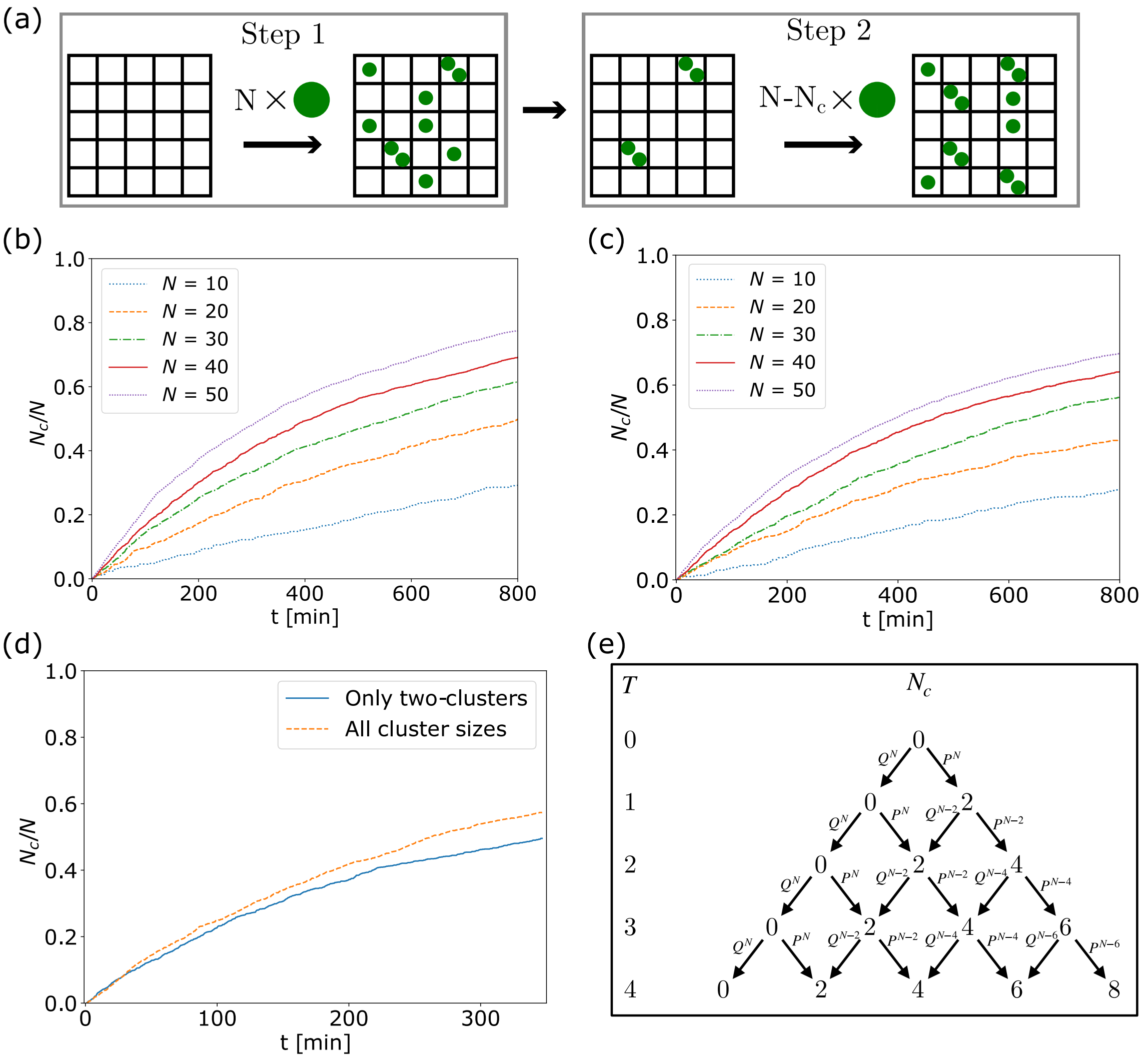

Supplemental Figure 2: **Balls-into-bins model.** (a) Sketch of the two steps in the balls-into-bins model. In Step 1 we assign each of the free particles (initially N particles) randomly to a box. In step 2 we remove all particles that were in a box by themselves and assign these again to a new box. (b) We present the results from the simulation of the balls-into-bins model for different initial values N. We used the parameters $D = 1200$, $S = 5$, $v_0 = 0.04$. In (c) we present the results of the dynamic simulations for the same number of active particles. In (d) we compare the results of the balls-into-bins simulation for moderately high concentrations ($N = 60$) when taking only two clusters into account with the simulation where all clusters are taken into account. The curves deviate for larger times but the deviation is relatively small. (e) Presents the first four steps of the tree diagram used for the derivation of the exact expression for the average number of particles in a cluster $\langle N_c \rangle$ in Eq. (3).

coefficient and we find

$$N_c(T,N) \approx \sum_{n=2, n\in 2\mathbb{Z}}^{2T} \left\{ n \binom{T}{n/2} P(N)^{\frac{n}{2}} Q(N)^{T-\frac{n}{2}} \right\} . \tag{4}$$

However, this expression is unbound, i.e., and $N_c \to \infty$ as $T \to \infty$ whereas Eq. (3) will plateau and $N_c \to N$ as $T \to \infty$.

Approximation as Poisson process We can find a simpler expression modelling the dynamics as a Poisson process. Again, we only consider two-clusters forming. To motivate the modelling as a Poisson process we first consider the case where N is constant, i.e., the number of balls one assigns to a box is kept constant at each step. This corresponds to Eq. (4). As the number of bins is very large, $B \gg 1$, and the probability of a cluster forming is very small, $N \ll B$, the binomial distribution converges to the Poisson distribution. That is, the expected number of clusters is linearly growing in time with the proportionality constant being given by the rate r at which clusters form. Here, the rate is given by the probability $P(N,B)$, i.e.,

$$\langle N_c \rangle = 2rT = 2P(N,B)T \tag{5}$$

Here the factor of two is due to $P(N,B)$ being the probability of a two-cluster forming and, as there are two particles in each cluster, the number N_c increases by two. For the low densities considered this is an excellent approximation of Eq. (4). A balls-into-bins simulation with a constant number of free particles results in such a linear relationship as well. Furthermore, for small T it is a good approximation of Eq. (3) as well. However, for high concentrations or long times the constant-rate Poisson approximation breaks down as it will grow without bound because it assumes a constant rate, i.e. a constant number of free particles. Thus, we have to consider a time-dependent rate. First, we can simplify the probability as

$$r \equiv P(N_f, B) = 1 - \frac{B!}{(B-N_f)! B^{N_f}} \approx 1 - \left(1 - \frac{N_f}{2B}\right)^{N_f - 1} \tag{6}$$

which for $B/N_f \gg 1$ is a very good approximation. Now, to include a time-dependent rate $r(T)$ we assume that the number of free particles is linearly decreasing in time with a rate set by $r(T)$, thus $N_f = N - 2rT$. We then find

$$r(T) = 1 - \left(1 - \frac{N - 2r(T)T}{2B}\right)^{N-2r(T)T-1} \tag{7}$$

which is an implicit expression for the rate r that does not have an explicit solution. However, as the rate of clusters forming is small we can Taylor expand the right-hand side to lowest order in r. After some straightforward simplifications, assuming $N \gg 1$ and $B \gg N_f$, we find for $\langle N_c(T) \rangle = 2r(T)T$ the simple expression

$$\frac{\langle N_c(T) \rangle}{N} \approx \frac{T}{\frac{B}{N} + T} . \tag{8}$$

This expression contains only a single free parameter, B/N, which is essentially the system size over particle number, i.e., the inverse density. This parameter is the time needed for half the particles to be part of a cluster, $\langle N_c(t = B/N) \rangle = N/2$. Note that at small times this reduces to the linear function

$$\frac{\langle N_c(T) \rangle}{N} \approx \frac{T}{B/N} \tag{9}$$

and thus we recover the Poisson process with constant rate. On the other hand, for large times we have $N_c(T) \to N$, and the function is bound.

1.4 Rescaling of the measured values for $(\tau_1^{260}/\tau_1^{180}/\tau_1^{90}/\tau_1^{0})$

We expect τ_1 to scale inversely proportional with the speed, as faster particles encounter each other faster and hence assemble more quickly. Secondly, faster sedimentation to the glass surface increases the number of single particles and hence increases τ_1. We also assume that this effect dominates over the faster increase in the particle density, as we use the density corrected value for τ_N, which is τ_1. These effects can be captured by rescaling the measured values for $\left(\tau_1^{260}/\tau_1^{180}/\tau_1^{90}/\tau_1^{0}\right)$ with the measured propulsion velocities for the two shapes (1.09, 0.78, 1.02

and 1.58 respectively) as well as with the inverse calculated sedimentation velocities ([?]).

Once a sedimenting crescents has reached its terminal sedimentation velocity the following equilibrium holds: $F_{drag} = F_g$. From the work of Tchen et al. [?] on the resistance experienced by particles with a similar shape, i.e. curved and elongated particles, we know that the fluid resistance, which here corresponds to the drag force F_{drag}, can be written as

$$\text{resistance} = F_{drag} = 2R\chi_0 * U * \zeta \tag{10}$$

with R the radius of the circle (see Fig. 1 of main text), the angle χ_0 which correspond to half the opening angle α, ζ the frictional coefficient and U the velocity of the flowing fluid which for a sedimenting particle is equal to the sedimentation velocity v_{sed}. Using these expressions we find for the sedimentation velocity

$$v_{sed} = \frac{m'g}{\zeta * 2R\chi_0} \tag{11}$$

where m' is the effective mass of the particle ($m' = m - V\rho$) and ζ can be written as [?]

$$\zeta = \frac{m\pi\eta}{\ln(l/b_0) + e} \tag{12}$$

with $\eta = 10^{-3}Pas$ the coefficient of viscosity, l the half-arclength of the bent rod and b_0 the cross-sectional radius at the center of the particle which corresponds to half the thickness at that point. The kinematic shape factor m and the dynamic shape factor e, which both depend on the angle χ_0, can be taken from [?].

For our 180° crescent with a thickness of $2\mu m$, a radius of $4\mu m$ and an arcleng of 4π, using $m_{180} = 3$ and $e_{180} = 0.1$ from Ref. [?], we find $v_{sed}^{180} = 1.14\mu m/s$. For our 90° crescent we obtain $v_{sed}^{90} = 0.73\mu m/s$ using $2b_0 = 1.5\mu m, R = \sin(\pi/4) * 8.5\mu m, L_{arc} = R * \pi/2, m_{90} = 3.6$ and $e_{90} = 0.8$ Ref. [?]. For a 260° crescent $v_{sed}^{260} = 0.95\mu m/s$ with $2b_0 = 2\mu m, R = 4\mu m, L_{arc} = R * 29/20\pi, m_{260} = 2.8$ and $e_{260} = -0.7$ Ref. [?]. Finally, for a straight rod $v_{sed}^{0} = 1.24\mu m/s$ with $2b_0 = 2\mu m, R = 4\mu m, L_{arc} = L = 10, m_0 = 4$ and $e_0 = 1.2$, see Ref. [?].

We can now use these sedimentation velocities as well as the measured propulsion velocities to rescale the measured values as follows:

$$\left(\frac{\tau_1^{180}}{\tau_1^{90}}\right)_c = \frac{\tau_1^{180} v_{prop}^{180} v_{sed}^{90}}{\tau_1^{90} v_{prop}^{90} v_{sed}^{180}} = \frac{2.87 \cdot 0.78 \cdot 0.73}{3.63 \cdot 1.02 \cdot 1.14} = 0.39 \tag{13}$$

$$\left(\frac{\tau_1^{180}}{\tau_1^{260}}\right)_c = \frac{\tau_1^{180} v_{prop}^{180} v_{sed}^{260}}{\tau_1^{260} v_{prop}^{260} v_{sed}^{180}} = \frac{2.87 \cdot 0.78 \cdot 0.95}{7.0 \cdot 1.09 \cdot 1.14} = 0.24 \tag{14}$$

$$\left(\frac{\tau_1^{180}}{\tau_1^{0}}\right)_c = \frac{\tau_1^{180} v_{prop}^{180} v_{sed}^{0}}{\tau_1^{0} v_{prop}^{0} v_{sed}^{180}} = \frac{2.87 \cdot 0.78 \cdot 1.24}{4.08 \cdot 1.58 \cdot 1.14} = 0.38 \tag{15}$$

This is in perfect agreement with the value found in simulations and implies that hydrodynamic attractions seem negligible.

2 Supplemental Discussion

2.1 Passive bend rod-shaped particles

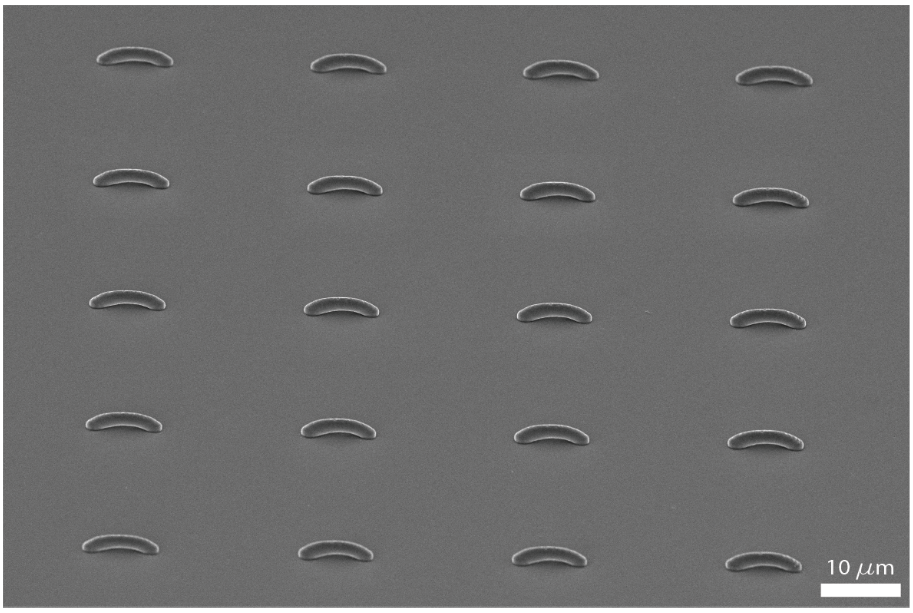

Supplemental Figure 3: Scanning electron microscopy image of an array of 3D printed bent rods with an opening angle of $\alpha = 90^{\circ}$.

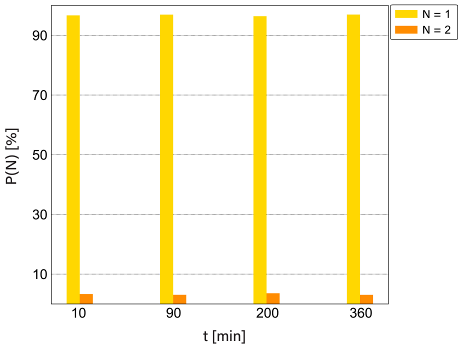

Supplemental Figure 4: Cluster distribution over time for a sample of passive 180° crescents suspended in water with a surface area fraction of ($\phi_s = 0.237\%$) after 90 min. This corresponds to a particle density of 94.4 crescents/mm^2 Passive crescents are not coated with a 5nm Pt-layer.

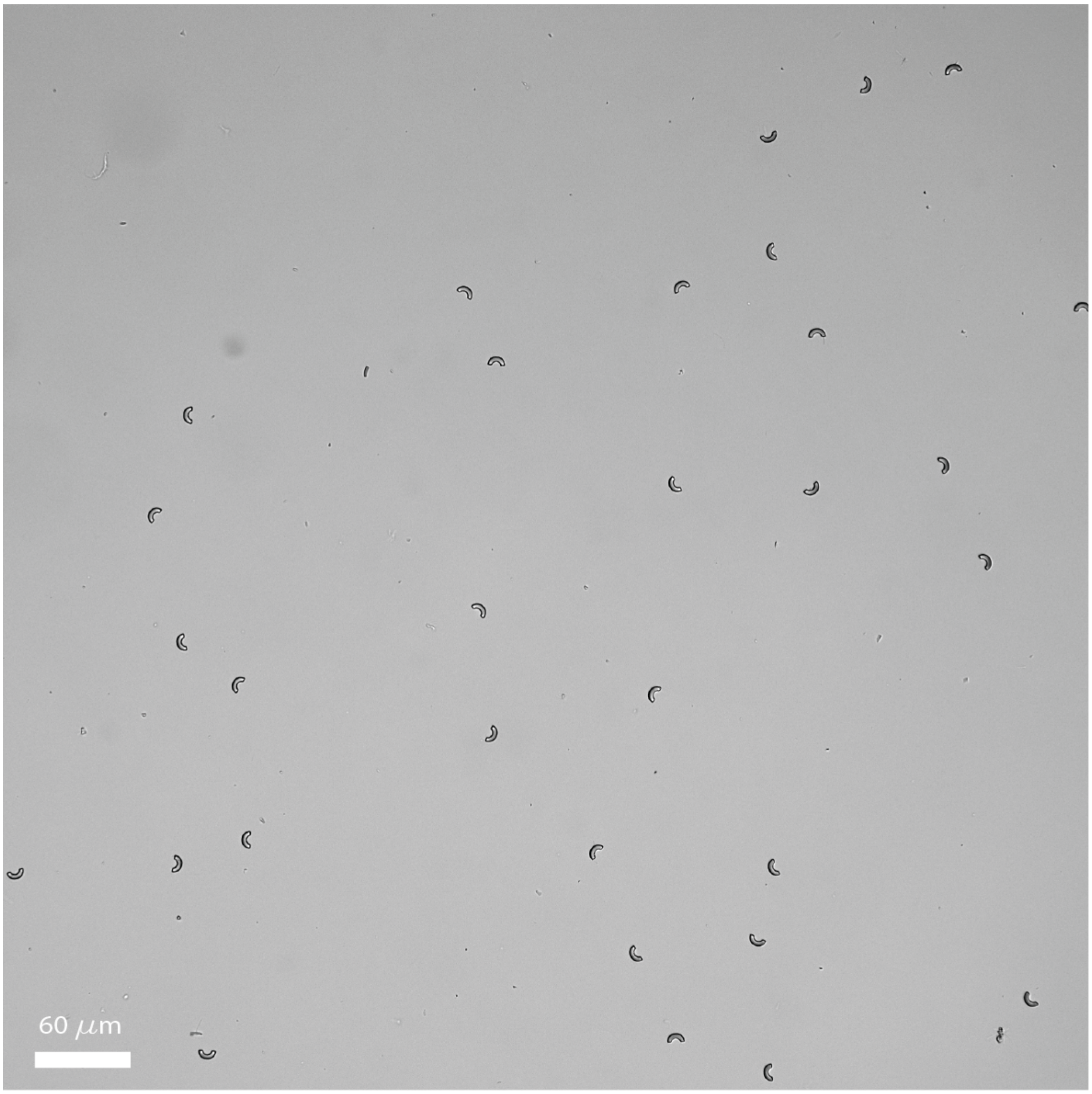

Supplemental Figure 5: Full field of view taken 360 min after suspending the particles in milliQ water ($\phi_s = 0.237\%$). Contrast and brightness were increased to improve the clarity of the image.

2.2 Self-organization of active concave-side leading 180° crescents

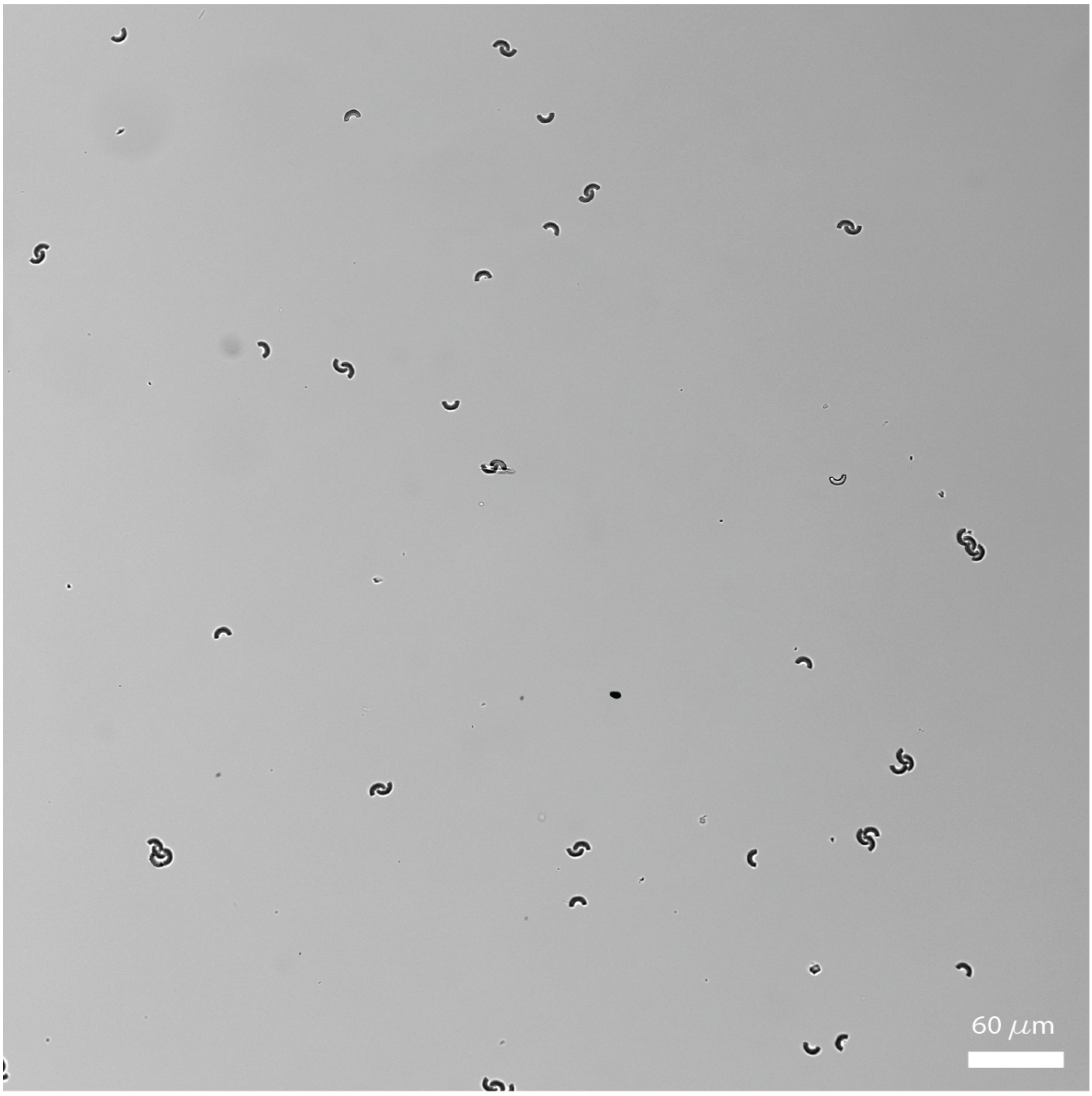

Supplemental Figure 6: Full field of view taken 90 min after mixing the particles and the fuel solution ($\phi_s = 0.263\%$). A detail of this field of view is shown in Fig. 2a. Contrast and brightness were increased to improve the clarity of the image.

2.3 30 sec trajectories of active concave-side leading 180° crescents

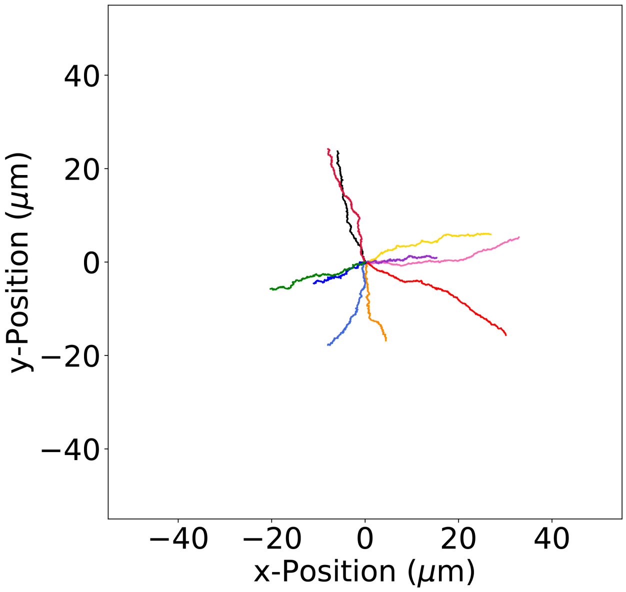

Supplemental Figure 7: 30 sec trajectories for concave-side leading 180° crescents taken ca. 5-20 min after mixing. Due to their size and shape, the motion of these active crescents shows long persistence lengths.The average particle velocity extracted from this data is $\langle v \rangle_{180} = 0.78 \pm 0.08\ \mu\mathrm{m\,s^{-1}}$.

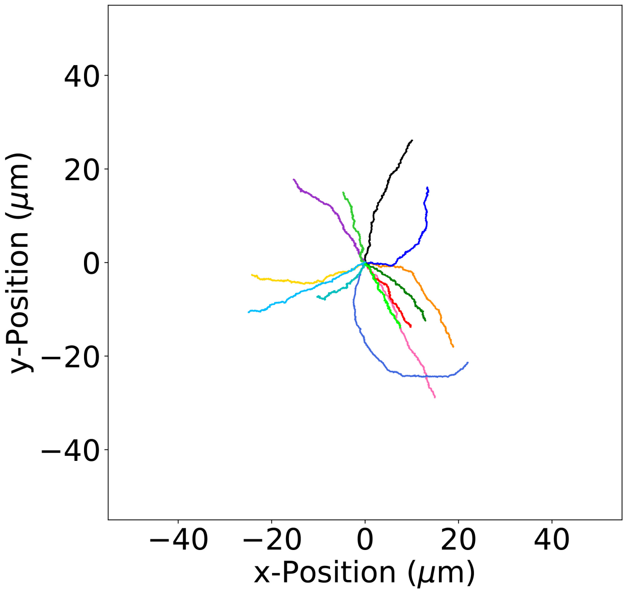

Supplemental Figure 8: 30 sec trajectories for concave-side leading 180° crescents taken ca. 5h after mixing. The average particle velocity extracted from this data is $\langle v \rangle_{180} = 0.81 \pm 0.02\ \mu\mathrm{m\,s^{-1}}$, and hence does not change significantly even after long times.

2.4 Cluster distribution and density profile for convex-side leading 180° crescents and straight rods

A decrease in particle density over time for convex-leading 180° crescents can be explained by the fact that these crescents sediment faster than their concave-leading counterparts. Since stuck single crescents are not taken into account when determining the total number of crescents and their number slowly increases in time, the overall density of particles decreases once all crescents have sedimented. This means that the fraction of clustered particles is overestimated when the particles density decreases.

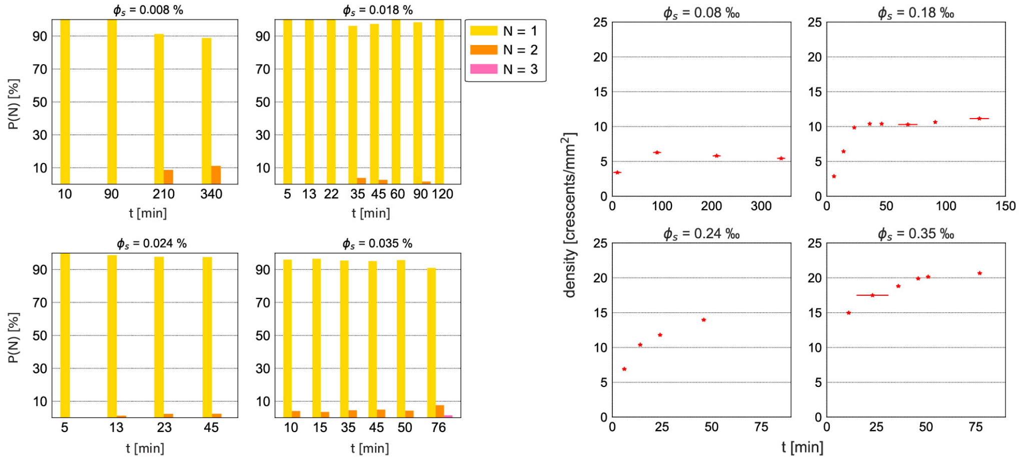

Supplemental Figure 9: Cluster size distributions (left) and corresponding density profiles over time (right) for samples of c^+ crescents with an opening angle of 180° and different surface area fractions ϕ_s. Samples correspond to the results shown in Figure 4h of the main text. Stuck single crescents are not taken into account when determining the total number of crescents.

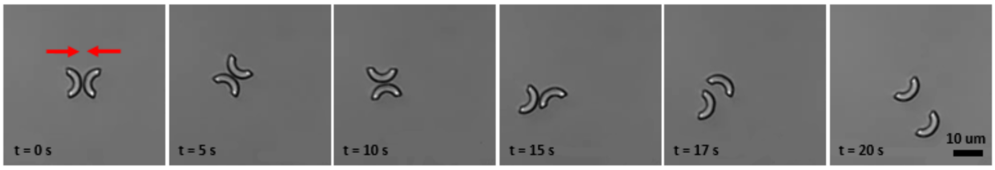

Supplemental Figure 10: Stills from Supporting Video4, showing that pairs of convex-side leading crescents destabilize by sliding past each other typically within min. The direction of motion is indicated by a red arrow.

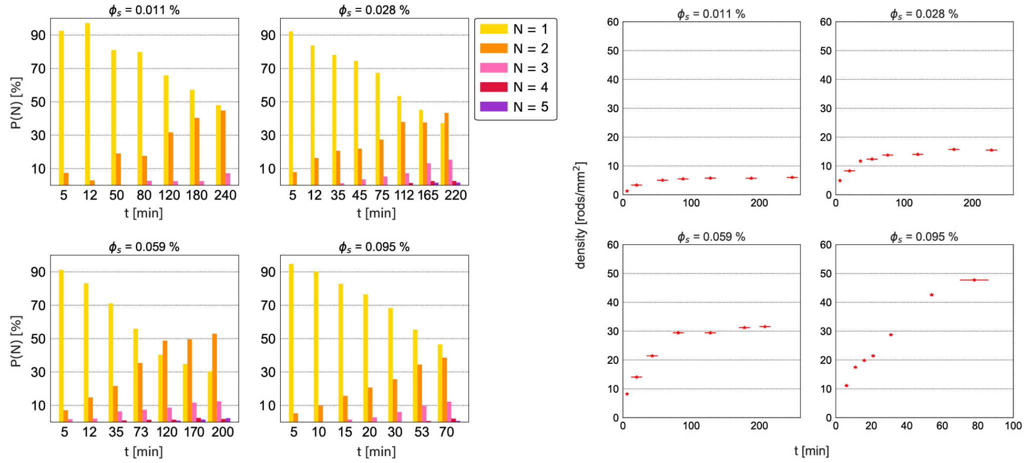

Supplemental Figure 11: Cluster size distributions (left) and corresponding density profiles over time (right) for samples of straight rods with different surface area fractions ϕ_s. Samples correspond to the results shown in Figure 4h of the main text. Stuck single rods are not taken into account when determining the total number of particles.

2.5 Cluster size distributions and density profiles for concave-side leading crescents

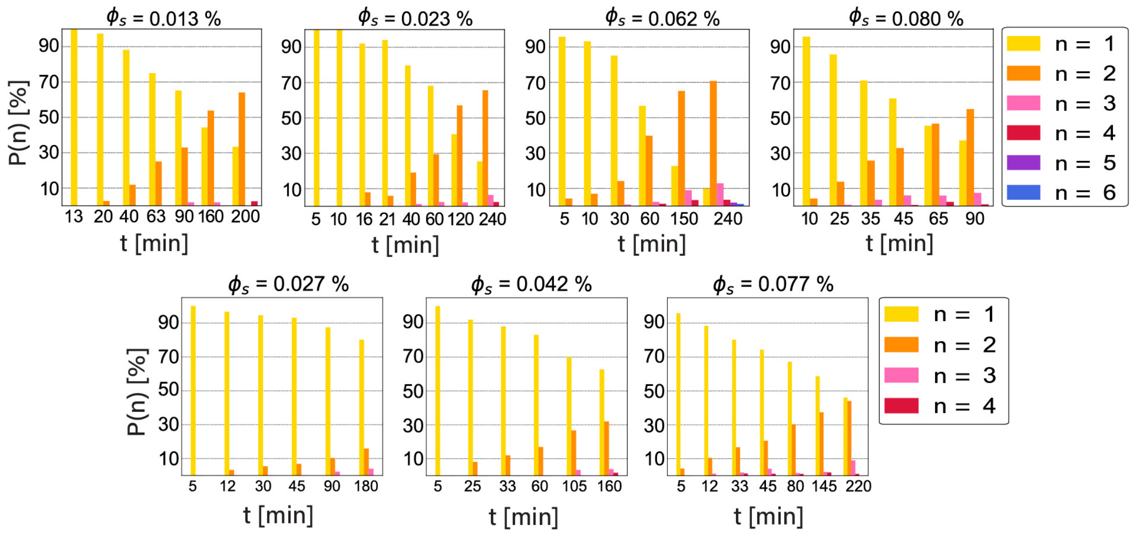

Supplemental Figure 12: Cluster size distributions over time for all samples of 90° (top) and 260° (bottom) concave-leading crescents mentioned in our work. Stuck single crescents are not taken into account when determining the total number of crescents.

It has to be noted that pairs of 260° crescents that are not properly interlocked but face each other with their ends touching, are also observed in significant amount in experiments. This is not the case in simulations and can be explained by the flat contact area that 3D-printed 260° crescents have at their ends in contrast to the round ends of simulated bend rods.

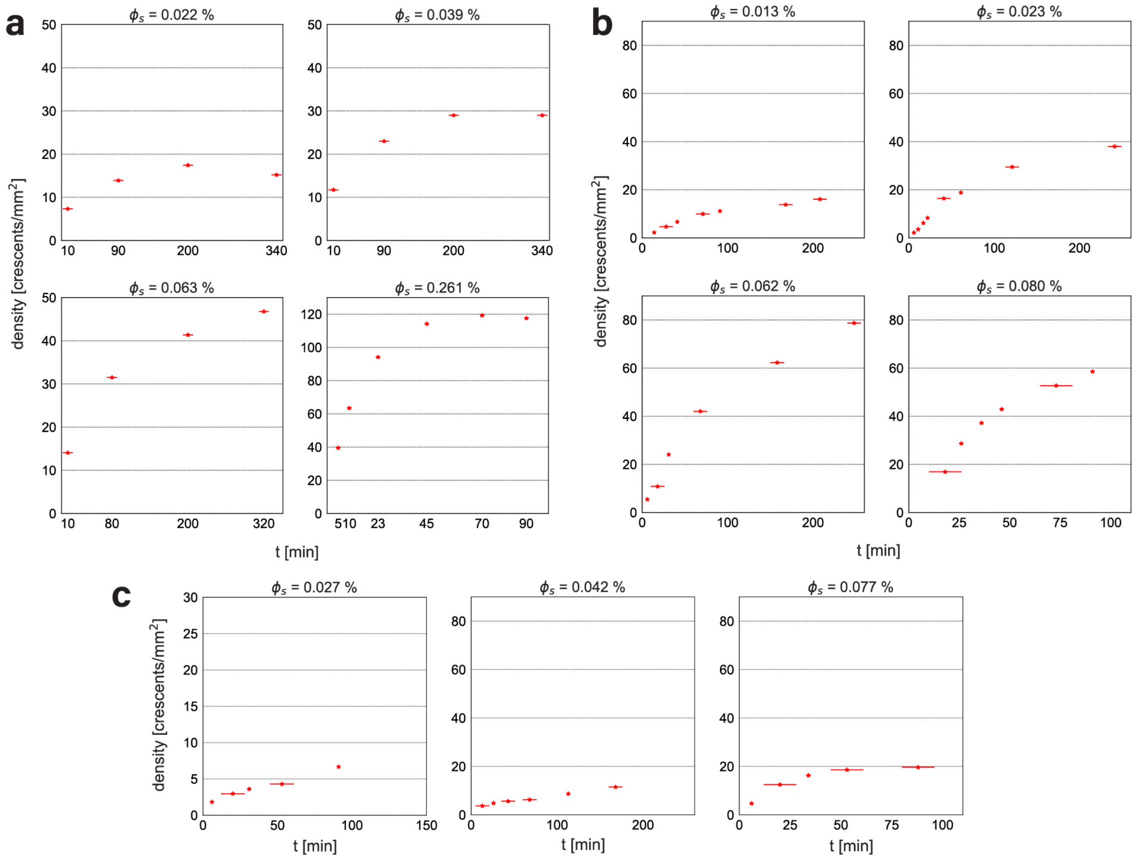

Supplemental Figure 13: Density profiles for all 180° (a), 90° (b) and 260° (c) concave-leading crescent-samples mentioned in our work. In all three cases the cross-section of the particles is 10 µm. Stuck crescents are taken into account in the density profiles.

2.6 Histograms for 180°-particles from simulations

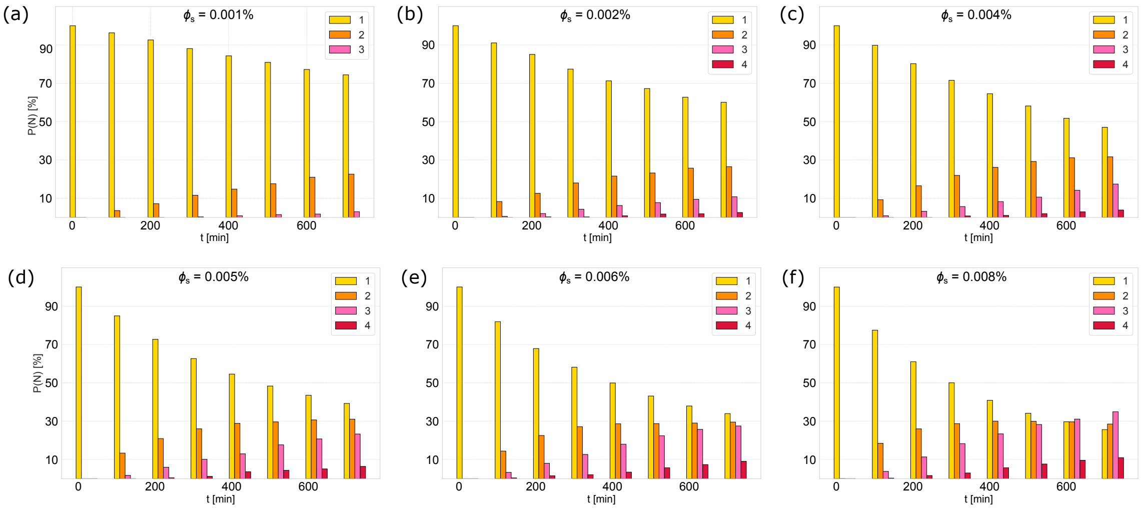

Supplemental Figure 14: **Histograms for 180°-particles from simulations**. We present the histograms of the cluster sizes over time for different concentrations. These are obtained from the dynamical simulations of the active particles with an opening angle of 180°.

2.7 Effect of noise

The simulations presented in the main text were performed in the limit where translational noise $\xi(t)$ and rotational noise $\eta(t)$ are vanishing (see Materials and Methods). To investigate the effect

of noise on the clustering dynamics we present here in Fig. 15 the results for different values of rotational noise. We choose delta-correlated translational and rotational noise, $\langle\xi(t)\xi(t')\rangle = 2D_t\delta(t-t')$ and $\langle\eta(t)\eta(t')\rangle = 2D_r\delta(t-t')$, with D_t and D_r the diffusion coefficients. We keep $D_t = 0.025$ μm/s constant and vary D_r in Fig. 15. We note that the experimental value for the rotational noise for 180° particles is found to be $D_r^{\text{exp}} \approx 0.005$ rad^2/s. We find that a noise of this order modifies the clustering dynamics only slightly. However, for much larger values of rotational noise the number of clustered particles at a given time decreases significantly.

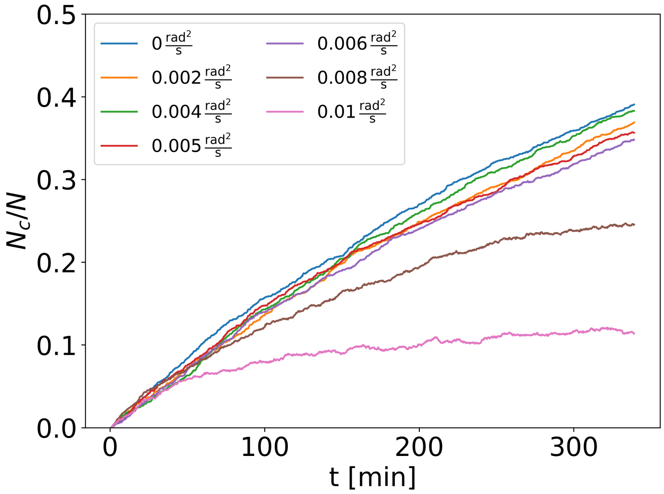

Supplemental Figure 15: **Rotational noise.** The clustering dynamics at $\phi = 0.005\%$ for 180° particles and for different values of the rotational noise D_r.

2.8 Time-dependence of the particle concentration

As explained in the main text, we find that the concentration in the experiments is not constant but increases initially while particles are sedimenting. We find that after a certain time the concentration plateaus and remains approximately constant afterwards. To investigate the large discrepancy of the clustering behavior we observe in experiments and simulations we consider a time-dependent concentration in the simulations as well. As described in the main text, if this is not taken into account we find that the active particles cluster significantly more and faster at a given concentration compared with the experiments. To this end, we measure experimentally how the concentration changes over time for different final concentrations. The results are presented in Fig. 13. We find that a square-root fit $N(t) = N(t=0) + b\sqrt{t}$, with two free parameters, approximates the experimental measurements well. We thus use these fits to obtain an expression for the concentration as function of time. This expression is then implemented in the simulation to increase the number of particles in the system over time as follows. While the concentration is less than the final concentration particles are added at the rate determined by the fit function. Once the final concentration is reached no more particles are added. This results in the $N(t)$ curves presented in Fig. 16a,b. Using these time-dependent concentrations we now turn towards studying the cluster dynamics. The results are presented in Fig. 16c-e. We now find a good quantitative agreement between experiments and simulations. This suggests that the time-dependent concentration was the major factor causing the large discrepancies between experiment and simulations mentioned in the main text. Note that the master curve we obtain (Fig. 16e) from the simulations is rather poor. The reason for this is found to be the naive implementation of the concentration increase. Namely, due to the hard cut-off once the final concentration, $N(t = t_{\text{fin}}) = N_{\text{fin}}$, is reached we find a small jump in the $N_c/N(t)$ curve exactly at the time t_{fin}. This can be seen in Fig. 16d. When computing the master curve, the size of this jump is exaggerated, see Fig. 16e. Thus, for times before the earliest cut-off the curves for different concentrations fall onto a single master curve. Note that this jump is not visible in the N_c curve in 16c and is purely due to the hard cut-off which enters in Figs. 16d,e when the y-axis is normalized by dividing by $N(t)$. Furthermore, note that this is a temporary effect. We find that for very long times the curves converge towards a single master curve once again.

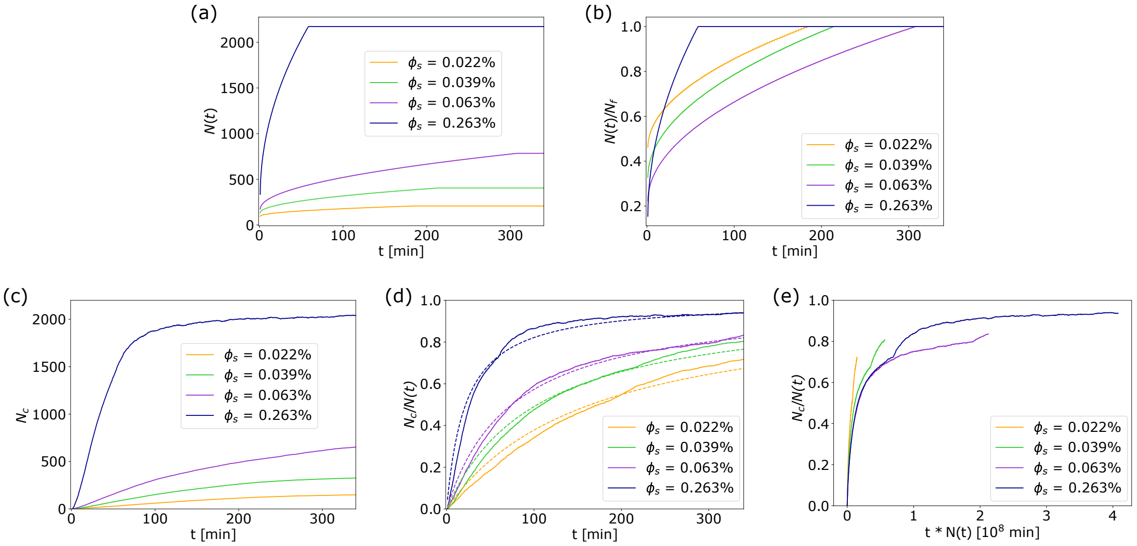

Supplemental Figure 16: **Time-dependent concentration in simulations.** Following experimental measurements we implement an increasing concentration in the simulations. The total number of particles in the system as a function of time for different final concentrations is shown in (a) and as a ratio of the final number of particles in (b). In (c) we show the total number of particles that are in a cluster (note the absence of significant jumps) while in (d) we show the relative number of particles. The solid lines are the data from simulations, the dashed lines best fits. In (e) we present the master curve.

2.9 Simulation results for bent rods with increasing opening angle

We present here the results from the simulations of the active particles with an opening angle from 30° to 330° and the same cross-sectional length as particles with an opening angle of 180°.

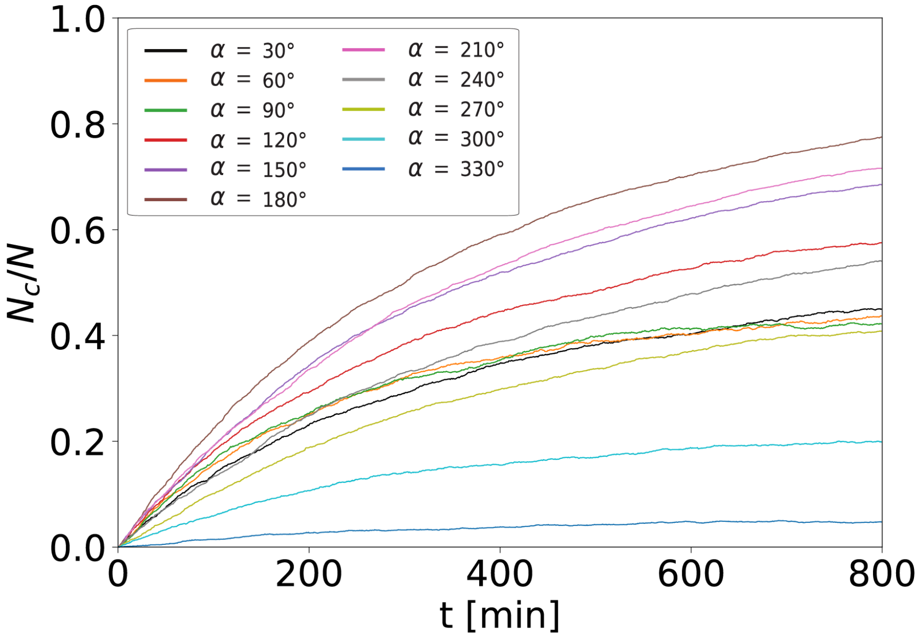

Supplemental Figure 17: Evolution of N_c/N over time for active crescents with fixed cross-sectional length ($L = 10$ µm) but different opening angles for $N = 66$ particles each.

2.10 Results for 90°-particles from simulations

We present here the results from the simulations of the active particles with an opening angle of 90° and the same arc-length as particles with an opening angle of 180° we simulated for Fig. 3 of the main text and Fig. 16. The results are thus different from the ones presented for simulations with an opening angle of 90° in the main text, where instead the *cross section* was kept the same as the 180°-particles. Here, the cross section of the 90°-particles is thus bigger than the one of the 180°-particles with the same arc-length. We find the clustering curves N_c/N presented in Fig. 18a. They are similar to the ones found for 180°-particles shown in Fig. 3d of the main text. The bigger cross-section of the 90° particles "compensates" for the smaller opening angle which, at the same cross-section, would result in clusters being more unstable. The corresponding master curve is shown in Fig. 18b. Finally, the cluster-size histograms for different concentrations are presented in Fig. 18c-g. The same quantities for 180°-particles of same arc-length are shown in Fig. 14.

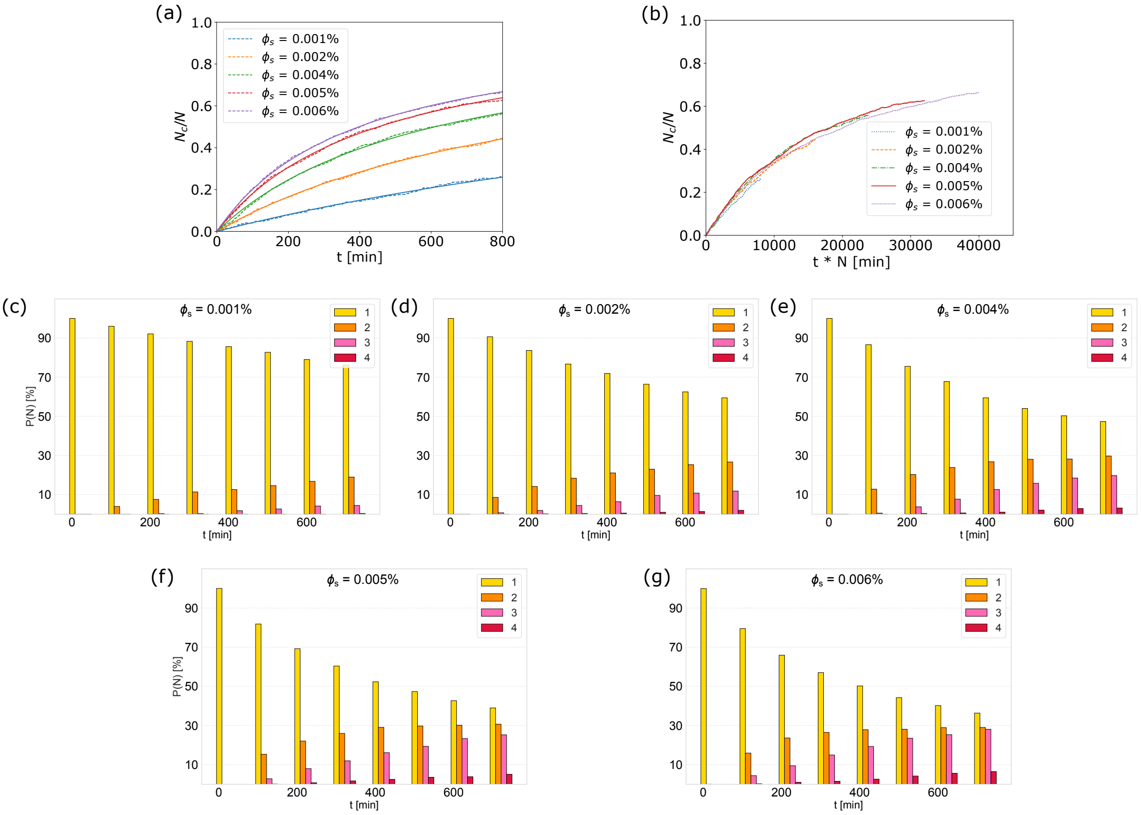

Supplemental Figure 18: **Results for 90°-particles from simulations.** (a) N_c/N curves for different concentrations for particles with an opening angle of 90° and (b) the corresponding master curve, found by rescaling time $t \to t * N$. In panels (c)-(g) we show the histograms for the size of clusters over time for the different concentrations.

2.11 Simulations for different opening angles with same arc-length

Here we present the results for simulations of particles with different opening angles when the arc-length of the particles is fixed. That is, the smaller the opening angle, the larger the cross-section of the particles. This is different of the results presented in the main text (Fig. 5) where the particles considered have instead the same cross-section. We find that over a large range of opening angles the clustering dynamics is quite similar, see Fig. 19. This is in stark contrast to the results for fixed cross-section where the clustering dynamics was significantly different for different opening angles.

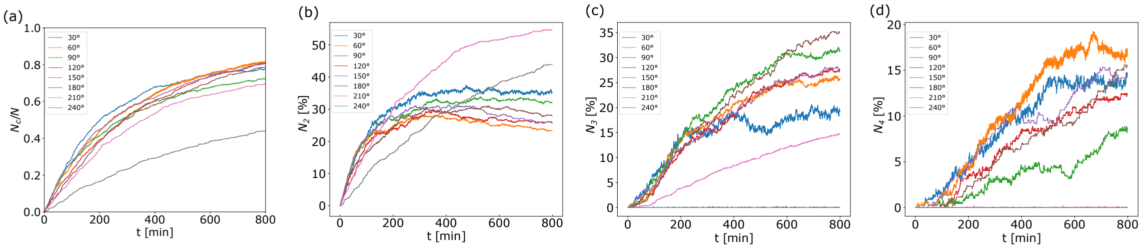

Supplemental Figure 19: **Simulations of particles with different opening angles but fixed arc-length**. (a) N_c/N curves versus time. Apart from the largest angle (240°) the curves for all angles considered are very similar. The percentage of two-, three-, and four-particle clusters over time is shown in panels (b)-(d), respectively. Large cluster sizes are rarely found.

2.12 Calculation of the active force and expected rotational diffusion constant

We can infer the active force from the measured velocity when bent rods are freely moving, through a force balance of the active force and drag: $F_{active} = F_{drag}$. With $F_{drag} = \zeta * v_{prop} * L_{arc}$ [?], where ζ is the frictional coefficient, v_{prop} the velocity and L_{arc} is the arclength. Using the values for v_{prop} and L_{arc} mentioned in section 1.4 as well as equation 12, we can directly calculate the active force as $F^0_{active} = 0.071pN$, $F^{90}_{active} = 0.041pN$,$F^{180}_{active} = 0.048pN$, and $F^{260}_{active} = 0.12pN$, where superscripts indicate the opening angle of the bend rods.

The angular velocity of a rotating crescent pair can be approximated in case of vanishing noise as:

$$\omega_{pair} = \frac{2F_{active} l D_r}{k_b T} \tag{16}$$

if we assume the translation-rotation coupling to be negligible [?]. For a crescent pair the effective lever arm l is 1/4 of the cross-sectional length L and the magnitude of the force vector corresponds to $2F_{active}$. For a rotating pair of 180° crescents with a typical angular velocity of $\approx 0.13\,\mathrm{rad\,s^{-1}}$ we can calculate the rotational diffusion constant D_r^{180} at 300K to be $2.2*10^{-3}s^{-1}$. This is comparable to the result obtained from calculations made with HYDRO++, a program for the calculation of hydrodynamic coefficients and other solution properties of colloidal particles, employing bead models [?]. The rotational diffusion constant for a crescent-pair-model made out of 16 beads is $D_r = 8.9*10^{-4}s^{-1}$ and the translation-rotation coupling coefficients are found to be several orders of magnitude lower than D_r which validates our assumption to neglect them.
