# Supplementary material for: Designing highly efficient interlocking interactions in anisotropic active particles
Source: Nat Commun. 2024 Jul 7;15:5692. doi: 10.1038/s41467-024-49955-x (PMC11227507; doi:10.1038/s41467-024-49955-x)
Supplement: Supplementary file 3 — Description of additional supplementary files [file 41467_2024_49955_MOESM3_ESM.pdf]

Description of the Videos

File Name: Supplementary Video 1
Description: Bright field microscopy video of a bent rod with an opening angle of 180° self-propelling in a 1% aqueous H_2O_2 solution. The particle moves concave-side leading. The original frame rate is 20 fps and the speed was enhanced 3x.

File Name: Supplementary Video 2
Description: Bright field microscopy video of a bent rod with an opening angle of 180° self-propelling in a 5% aqueous H_2O_2 solution. The particle moves convex-side leading. The original frame rate is 20 fps and the speed was enhanced 3x.

File Name: Supplementary Video 3
Description: Bright field microscopy video of a bent rod with an opening angle of 90° self-propelling in a 1% aqueous H_2O_2 solution. The particle moves concave-side leading. The original frame rate is 20 fps and the speed was enhanced 3x.

File Name: Supplementary Video 4
Description: Bright field microscopy video showing the formation of a 180° crescent-pair in a suspension of concave-side leading particles (1% H_2O_2). Cluster exhibits rotational motion and almost no translational motion. The original frame rate is 44 fps and the speed was enhanced 3x.

File Name: Supplementary Video 5
Description: Bright field microscopy video showing that larger clusters of concave-side leading 180° crescents (1% H_2O_2) also exhibit rotational motion and almost no translational motion. The original frame rate is 32 fps and the speed was enhanced 3x.

File Name: Supplementary Video 6
Description: Bright field microscopy video showing that larger clusters of concave-side leading 180° crescents (1% H_2O_2) also exhibit rotational motion and almost no translational motion. The original frame rate is 44 fps and the speed was enhanced 3x.

File Name: Supplementary Video 7
Description: Bright field microscopy video showing the formation of a 90° crescent-pair in a suspension of concave-side leading particles (1% H_2O_2). The original frame rate is 20 fps and the speed was enhanced 3x.

File Name: Supplementary Video 8
Description: Bright field microscopy video showing two rotating pairs of straight rods. When the particles are not perfectly aligned, the pair will be less stable against break-up due to the lack of interlocking. The original frame rate is 20 fps and the speed was enhanced 3x.

File Name: Supplementary Video 9
Description: Bright field microscopy video showing the breaking of a pair of straight rods. Straight rods have no ability to interlock. The original frame rate is 20 fps and the speed was enhanced 3x.

File Name: Supplementary Video 10
Description: Bright field microscopy video showing two rotating pairs of crescents with an opening angle of 260°. Once formed, clusters exhibit significant stability due to their highly efficient interlocking. The original frame rate is 20 fps and the speed was enhanced 3x.

File Name: Supplementary Video 11
Description: Bright field microscopy video showing that for crescents with an opening angle of 260° the probability for cluster formation is low, due to their relatively small opening length. The original frame rate is 20 fps and the speed was enhanced 3x.

File Name: Supplementary Video 12
Description: Bright field microscopy video showing that the few pairs observed over the course of the experiment for convex-side leading 180° crescents (5% H_2O_2) usually were not stable beyond max. 2 min. The original frame rate is 173 fps and the speed was enhanced 3x.

File Name: Supplementary Video 13
Description: Results from a simulation of a small system and high density to illustrate the cluster formation process for 180° crescents. See the main text and the SM for details on the simulations. According to the mapping described in the SM, one second of the video corresponds to ≈ 4.8s of physical time.
